# Supplementary material for: Rapid identification of Biomphalaria spp. and diagnosis of Schistosoma mansoni infestation using MALDI-TOF mass spectrometry
Source: PLoS Negl Trop Dis. 2026 Mar 24;20(3):e0013236. doi: 10.1371/journal.pntd.0013236 (PMC13056252; doi:10.1371/journal.pntd.0013236)
Supplement: Attachment — Submitted filename: sample-response-reviewers.docx [file pntd.0013236.s001.docx]

**Manuscript ID: PNTD-D-25-00946**

**Response to Reviewers**

Thank you for allowing us to submit a revised version of our manuscript to *PLOS NTD.* We appreciate the time and effort that you and the reviewers dedicated to providing feedback on our manuscript and are grateful for the insightful comments and valuable improvements to our paper. We have incorporated in the revised version all the suggestions made by the reviewers. Those changes are highlighted within the manuscript. Please see below, in blue, for a point-by-point response to the reviewers’ comments and concerns. All page numbers refer to the revised manuscript file with tracked changes. We hope that this enhanced version will comply with the *PLOS NTD* publication standards.

**Reviewers' Comments to the Authors:**

**Reviewer 1**

**Author response: Thank you!**

1. The snails were infected in a controlled laboratory environment, which may not fully represent the natural dynamics of infection in nature.

**Author response**: This study is a first approach to confirm the ability of MALDI-TOF MS to discriminate between *Biomphalaria* infested with the parasite *Schistosoma mansoni* (*S. mansoni*) and those with any infestation. We agree that controlled laboratory environment differ from the natural dynamics of infection in nature. In nature, snails (*Biomphalaria*) are infested by several parasites at different stages. This does not allow for harmonization or the establishment of a database specific to *S. mansoni* infestation. After the databases has been established, snails can be collected in the field and their MALDI-TOF spectra compared to those in the database.

2. The study did not address how the infection stage or parasite load affects protein profiles detected by MALDI-TOF MS.

**Author** **response**: The individual (samples) in our study were infected by five *S. mansoni* eggs on the same day under the same conditions. The parasite load was measured by real-time PCR between day 21 and day 30 of infestation. However, there was no significant differences in the MALDI-TOF spectra, despite differences in the CTs obtained.

3. There is currently no established MALDI-TOF MS reference spectra database for *Schistosoma* parasites or infected snails, which is necessary for wider diagnostic use.

**Author response:** The objective of this work was to setup a MALDI-TOF reference spectra database including *Biomphalaria* sails infested or not by *S. mansoni*. This database can be made available for research or epidemiological purposes upon request.

**Reviewer 2**

**Author response: Thank you!**

**Reviewer 3**

**Author response: Thank you!**

**Reviewer 4**

**Author response: Thank you!**

1. Yes, the results were well presented and consistent with the study design. The figures were of sufficient quality and clarity. Could the author include MALDI-TOF MS profiling with spectra and a dendrogram for both uninfected and S. mansoni-infected snails?

**Author response**: The Figure 3 illustrates the PCA comparison of the spectra obtained from the two Biomphalaria species, whether infected or not infected by *S. mansoni*. Please find below the dendrograms of each *Biomphalaria* species tested, as requested.
