# Supplementary material for: Rapid identification of Biomphalaria spp. and diagnosis of Schistosoma mansoni infestation using MALDI-TOF mass spectrometry
Source: PLoS Negl Trop Dis. 2026 Mar 24;20(3):e0013236. doi: 10.1371/journal.pntd.0013236 (PMC13056252; doi:10.1371/journal.pntd.0013236)
Supplement: Attachment — Submitted filename: response-reviewers-2b.docx [file pntd.0013236.s002.docx]

Dear Editor,

Thank you for allowing us to submit a revised version of our manuscript entitled “Rapid identification of Biomphalaria spp. and diagnosis of Schistosoma mansoni infestation using MALDI-TOF mass spectrometry” to PLOS NTD. We appreciate the time and effort that you and the reviewers dedicated to providing feedback on our manuscript and are grateful for the insightful comments and valuable improvements to our paper. We were sorry to hear that we have not responded to the concerns raised by Reviewer 3. Regrettably, we could not find any specific remark regarding the initial version that he made. There were no further remarks and comments from the reviewers we had to address.

In this revised version, we have shortened the snail collection and laboratory infection section because we refer to a recent paper, in which it has been fully described. All these changes are highlighted in the marked-up copy of the manuscript.
